# Optimal ultrasonication process time remains constant for a specific nanoemulsion size reduction system

**DOI:** 10.1038/s41598-021-87642-9

**Published:** 2021-04-29

**Authors:** Anubhav Pratap-Singh, Yigong Guo, Sofia Lara Ochoa, Farahnaz Fathordoobady, Anika Singh

**Affiliations:** 1 Faculty of Land and Food Systems (LFS), The University of British Columbia, Vancouver Campus 213-2205 East Mall, Vancouver, BC V6T 1Z4 Canada; 2 Department of Chemistry and Nanotechnology, Monterrey Institute of Technology and Higher Education (ITESM), Monterrey Campus, Av. Eugenio Garza Sada 2501 Sur, Tecnologico, 64849 Monterrey, N.L. Mexico

**Keywords:** Chemical engineering, Nanobiotechnology, Nanoparticles

## Abstract

This paper theorizes the existence of a constant optimum ultrasound process time for any size-reduction operation, independent of process parameters, and dependent on product parameters. We test the concept using the case of ‘ultrasonic preparation of oil-in-water nanoemulsions’ as model system. The system parameters during ultrasonication of a hempseed oil nanoemulsion was evaluated by a response surface methodology, comprising lecithin and poloxamer-188 as surfactants. Results revealed that the particle size and emulsion stability was affected significantly (*p* < 0.05) by all product parameters (content of hempseed oil-oil phase, lecithin and polaxamer-surfactants); but was not significantly (*p* > 0.05) affected by process parameter (‘ultrasonication process time’). Next, other process parameters (emulsion volume and ultrasonic amplitude) were tested using kinetic experiments. Magnitude of particle size reduction decreased with increasing ‘ultrasonication process time’ according to a first order relationship, until a minimum particle size was reached; beyond which ultrasonication no longer resulted in detectable decrease in particle size. It was found that the optimal ultrasonication process time (defined as time taken to achieve 99% of the ‘maximum possible size reduction’) was 10 min, and was roughly constant regardless of the process parameters (sample volume and ultrasonic amplitude). Finally, the existence of this constant optimal ultrasonication process time was proven for another emulsion system (olive oil and tween 80). Based on the results of these case studies, it could be theorized that a constant optimum ultrasonication process time exists for the ultrasonication-based size-reduction processes, dependent only on product parameters.

## Introduction

Nanoemulsions (NEs) are translucent or cloudy, thermodynamically unstable, and isotropic liquid mixtures of oil and water, often stabilized by a surfactant or a combination of a surfactant-cosurfactant^1^. Thanks to their high surface area, long shelf life, transparent appearance and tunable rheology, nanoemulsions have been applied across many fields such as drug delivery and food fortification over the past decades^2–4^. Using nanoemulsions helps hydrophobic drugs dissolve in the water phase and protects protein compounds through gastrointestinal tract^5^. Such emulsions can also be used to develop beverages loaded with lipophilic nutraceuticals, like encapsulated β-carotene, curcumin or insulin^2,3,6^, lipophilic hormones like insulin^7^, or as precursors to preparation of encapsulated microcapsules^8,9^. A high bioavailability for such emulsion systems can be achieved by ensuring small size of the dispersed droplets. This is often made possible by imposing very high shearing stresses upon the liquid that is to be dispersed, wherein the shearing forces break the material into multiple fine particles.

A widely used high energy method to reduce the droplet size of nanoemulsions is ultrasonication. In this method, mechanical vibrations from ultrasound waves (> 20 kHz) create sinusoidal pressure variation in the emulsion system^10^. This processing leads to microjet and shock-wave impacts and collisions between particles, resulting in particle-size reduction^11^. Even though it is essential to know the particle size once it reaches equilibrium during the ultrasonication process, it is vital to understand the dynamic pathways to reduce the processing time and to avoid the over-supply of energy, which may result in higher particle size than expected^12^. Based on previous study^11^, the droplet size first decreases exponentially with increasing ultrasonication time, then tended to be stationary after certain minutes. Thus, it is not necessary to keep increasing processing time to get the smallest particle size^11^. It has also been proved that the particle size decreasesd with ultrasonication time but was insensitive to ultrasonication amplitude^10^. Besides, based on the laws of Rittinger, Bond and Kick, the reduction of particle size is independent of the sample volume^13^. Hence, for reducing process time and energy during ultrasonication based nanoemulsion production, this article focuses on bridging the gap amongst these theories and introducing the concept of existence of very similar optimal ultrasound processing times for samples with different volumes produced by various ultrasonic amplitudes.

In this study, hemp seed oil (HSO) was selected as the oil phase to produce oil-in-water (O/W) nanoemulsions. HSO is the extracted oil from the seed of *Cannabis sativa L*. The presence of γ-linolenic acid, stearidonic acid, tocopherols, tocotrienols, phospholipids, carotenes, minerals as well as terpenoids and β-sitosterols provides an oil phase with superior nutritional value compared to other plant oils^14–19^. The combination of lecithin and poloxamer 188 (with HLB of 4 and 29, respectively) was selected as the surfactant of the nanoemulsion system. The contents of two surfactants and the hemp seed oil were optimized by using response surface methodology (RSM), a collection of statistical and mathematical techniques suitable for developing, improving, and optimizing processes^20,21^. Most importantly, the relationship between processing time and particle size was first fitted into well-known mathematical models. A constant optimal ultrasound processing time for different samples was also first confirmed. The theory of existence of this constant ultrasound processing time was also confirmed for another emulsion system using olive oil and Tween-80.

## Results and discussion

### Preparing condition optimization

The experimental results for the particle sizes (nm) using the central composite design are shown in Table 1. The four factors including hemp seed oil content (%), poloxamer 188 content (%), lecithin content (%) and processing time (min) were optimized using response surface methodology (RSM), and the results of the standard analysis of variance (ANOVA) are also represented in Table 1. The regression equation for the particle size as a function of hemp seed oil content (A, % v/v), poloxamer 188 content (B, % v/v) lecithin content (C, % w/v) and processing time (D, min) is given as Eq. (1).

1 $$\begin{aligned} {\text{Particle}}\;{\text{size}}\left( {{\text{nm}}} \right) & = {317}{-}{8}.0{\text{A}}{-}{51}.{\text{7B}}{-}{14}.{\text{9C}} + {1}.{9}0{\text{D}} + {1}.{\text{34A}}^{{2}} + {2}.{\text{22 B}}^{{2}} + {6}.{\text{12C}}^{{2}} \\ & \quad + 0.{\text{33 D}}^{{2}} { + 5}.{\text{88AB}}{-}{2}.{\text{78AC}}{-}0.{\text{89AD}}{-}{1}.{\text{64 BC}}{-}0.{\text{41BD}}{-}{1}.{\text{22 CD}} \\ \end{aligned}$$

**Table 1:** Central Composite Rotatable Design (CCRD) and corresponding ANOVA analysis.

Std Order	Factor 1 A:Hemp seed oil (% v/v)	Factor 2 B:Poloxamer (% v/v)	Factor 3 C:Lecithin (% w/v)	Factor 4 D:Time (min)	Particle size (nm)
1	5	1	0	5	263.9
2	10	1	0	5	362.8
3	5	5	0	5	253.1
4	10	5	0	5	383.6
5	5	1	5	5	212.5
6	10	1	5	5	280
7	5	5	5	5	208.3
8	10	5	5	5	283.7
9	5	1	0	20	423.1
10	10	1	0	20	343.8
11	5	5	0	20	277.8
12	10	5	0	20	505.5
13	5	1	5	20	294.1
14	10	1	5	20	210.9
15	5	5	5	20	179.9
16	10	5	5	20	220.4
17	7.5	3	2.5	12.5	201.8
18	7.5	3	2.5	12.5	202.6
19	7.5	3	2.5	12.5	192.4
20	7.5	3	2.5	12.5	200.3
21	2.5	3	2.5	12.5	162.9
22	12.5	3	2.5	12.5	274.7
23	7.5	0	2.5	12.5	223.7
24	7.5	7	2.5	12.5	198.7
25	7.5	3	0	12.5	332.1
26	7.5	3	7.5	12.5	194
27	7.5	3	2.5	0	345.5
28	7.5	3	2.5	27.5	175.4
29	7.5	3	2.5	12.5	206.6
30	7.5	3	2.5	12.5	191.8

ANOVA analysis of the CCRD model					
Source	df	Sum of squares	Mean square	*F*-value	*p*-value Prob < *F*
Model	15	160,550	10,703.3	3.67	0.007
A-HSO	1	20,510	20,510.1	7.12	0.018
B-Poloxamer	1	691	691.2	0.36	0.559
C-Lecithin	1	60,000	60,000.0	25.68	0.000
D-Time	1	733	733.6	0.31	0.568
A^2^	1	5493	5493.4	0.69	0.419
B^2^	1	4117	4117.4	0.53	0.479
C^2^	1	17,438	17,438.4	7.95	0.014
D^2^	1	16,551	16,550.9	2.55	0.132
AB	1	13,818	13,818.0	4.80	0.046
AC	1	4816	4816.4	1.67	0.217
AD	1	4442	4442.2	1.54	0.235
BC	1	1082	1082.4	0.38	0.550
BD	1	598	597.8	0.21	0.656
CD	1	8372	8372.2	2.91	0.110

The *F*-value of 3.67 along with the value of “Prob > *F*-value” of 0.007 (< 0.01) implied the model was significant and it is only a 0.7% chance that an *F*-value at this value could occur due to noise (Table 1). The terms A, C, C^2^, and AB (*p* < 0.05) were the statistically significant terms in this mathematic model. A signal (response) to noise (deviation) ratio of 7.153 was obtained in this study, which indicated an adequate precision for the signal (a value greater than 4 is desirable) and therefore, the model could be used to navigate the design space. For better understanding, the relation and effects of process factors on response variables are presented in Supplementary Fig. S1a as 3-d response surface plots.

**Figure 1:**
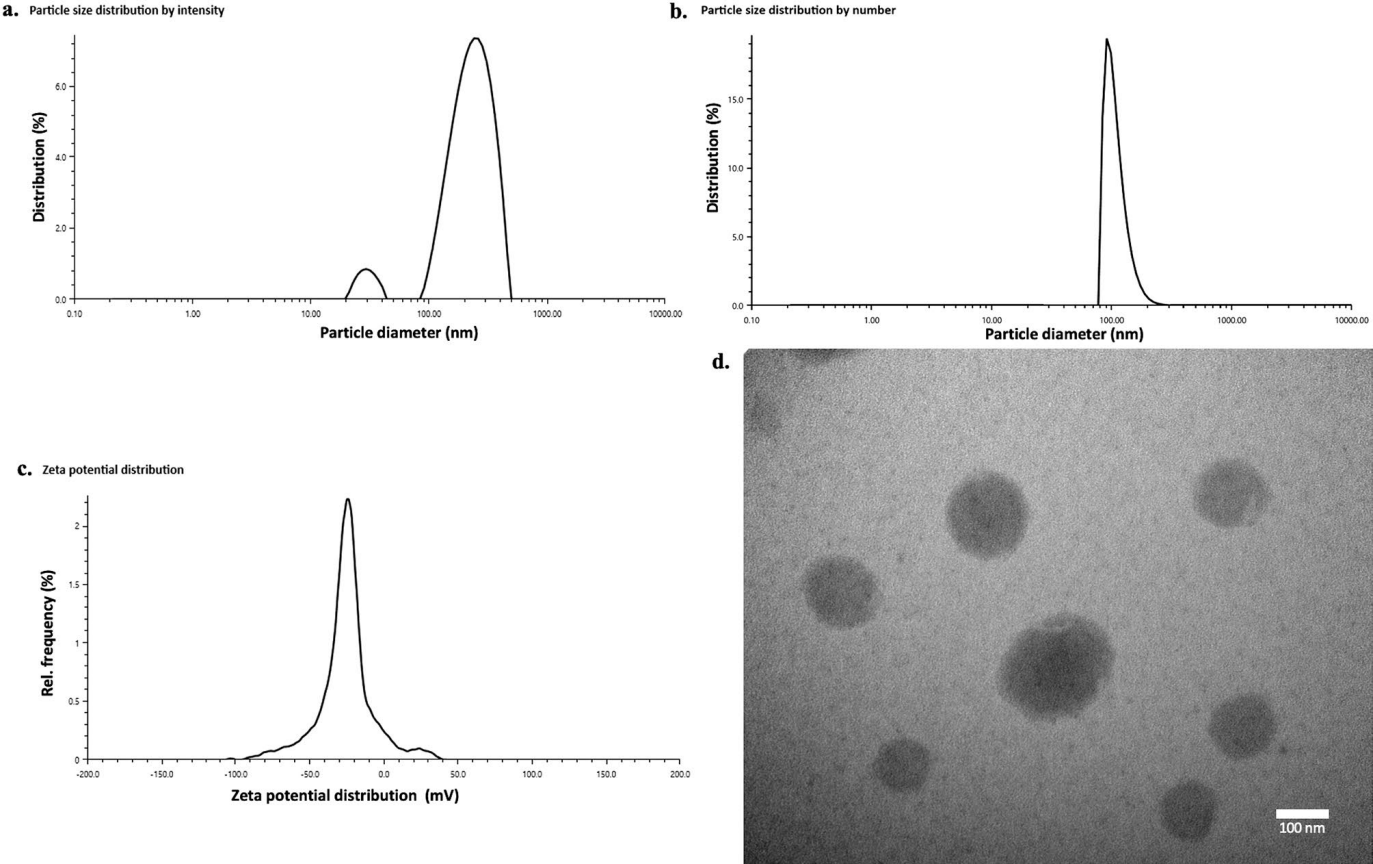
Optimal nanoemulsion characterization (particle size distribution; zeta potential distribution; and TEM images).

Using graphical and numerical optimization, the optimum condition for preparation was evaluated based on minimization of the hydrodynamic diameter (nm). The overall optimal condition resulted in particle size of 182.6 nm and was predicted to be achieved at combined levels of 12.5% (v/v) hemp seed oil, 2.6% (v/v) poloxamer 188, 5.9% (w/v) lecithin and 21.7 min of ultra-sound treatment with the composite desirability of 0.985 (Supplementary Fig. S1b). The optimal levels of parameters were verified by an additional verification experiment and resulted in emulsion droplets of average diameter 176.4 nm (Fig. 1). As minor signal where the size distribution was correlated with the light scattering intensity might give strong fraction presented on the histogram size by number^24^. To ensure the accuracy of the result given by DLS, both measurements of DLS (number by size and size distribution by intensity) were given in this stuty. For this processing condition, the Polydispersity index (PDI), zeta potential and entrapment efficiency were also tested which were 0.239, 31.4 mv and 96.08%, respectively. The TEM investigation of the optimal emulsion showed its spherical appearance with uniform size (Fig. 1). Besides, In Table 1, it can be found that some results showed smaller particle sizes compared with optimal result such as 162.9 nm and 175.4 nm. These were the results of outside points used to estimate the curvature of the response surface. Therefore, these results above indicated that the optimal processing condition was highly suitable for this nanoemulsion system.

### Emulsion characterization

The zero-order, first-order and second-order models are the most common and well-understood mathematical models used for modeling in chemical engineering investigations^9,22,23^. The obtained particle sizes reduction of nanoemulsions at various volumes (mL) for a period of 1 h were fitted to these models respectively and the fitted parameters are provided in Table 2. Amongst all the models tried in this study, the first-order model gave the best fit (mean R^2^ = 0.95 ± 0.04). In contrast, the mean R^2^ for the zero-order and second-order models were 0.39 ± 0.14 and 0.62 ± 0.12 respectively, which means they did not fit as well as the first-order model.

**Table 2:** Parameters of the three mathematical models tested.

Model		Zero-order model			First-order model				Second-order model			
Volume/mL	Amplitude/%	a	b	R^2^	a	b	c	R^2^	a	b	c	R^2^
	25	− 1.481 ± 1.573	336.0 ± 38.8	0.3058	162.8 ± 11.0	0.2718 ± 0.0774	270.0 ± 4.2	0.9910	143.5 ± 81.4	− 9962 ± 80	299.6 ± 29.2	0.6528
5	50	− 3.941 ± 3.523	340.3 ± 87.2	0.3832	375.6 ± 37.1	0.2819 ± 0.0945	156.4 ± 15.1	0.9813	331.9 ± 204.6	− 9928 ± 10,133	247.6 ± 73.9	0.6120
	100	− 2.433 ± 2.211	302.6 ± 54.7	0.3753	238.6 ± 57.2	0.2067 ± 0.1315	173.9 ± 18.8	0.9146	227.5 ± 104.5	− 10,020 ± 440	243.5 ± 37.3	0.7391
	25	− 3.857 ± 3.064	366.2 ± 75.8	0.4404	326.6 ± 39.2	0.2982 ± 0.0861	189.9 ± 18.1	0.9724	288.6 ± 199.9	− 9938 ± 1203	278.5 ± 71.7	0.5550
10	50	− 3.422 ± 4.04	395.4 ± 99.9	0.2627	404.0 ± 21.1	0.4300 ± 0.0573	261.8 ± 8.2	0.9946	354.4 ± 208.3	− 9922 ± 7867	309.4 ± 74.9	0.6342
	100	− 3.704 ± 3.675	401.0 ± 90.9	0.3352	393.9 ± 42.3	0.2241 ± 0.1415	201.8 ± 15.7	0.9774	364.3 ± 171.1	− 9816 ± 6136	309.6 ± 60.9	0.7301
	25	− 4.475 ± 3.069	411.7 ± 75.9	0.5136	340.7 ± 61.0	0.2211 ± 0.1159	189.2 ± 29.7	0.9396	312.7 ± 212.6	− 9844 ± 7786	311.8 ± 76.7	0.5646
20	50	− 3.910 ± 4.241	375.3 ± 104.9	0.2967	439.9 ± 29.4	0.3560 ± 0.0811	208.7 ± 11.1	0.9912	399.7 ± 203.2	− 9826 ± 12,980	277.5 ± 72.7	0.6981
	100	− 3.013 ± 3.122	380.7 ± 77.2	0.3163	326.3 ± 27.6	0.3291 ± 0.0995	251.6 ± 10.5	0.9860	290.2 ± 160.6	− 9874 ± 5280	306.8 ± 56.7	0.6605
	25	− 5.011 ± 3.225	448.0 ± 79.7	0.5450	367.6 ± 78.7	0.1880 ± 0.1413	166.8 ± 37.8	0.9157	337.9 ± 232.4	− 9909 ± 12,230	337.2 ± 83.0	0.5582
30	50	− 3.910 ± 2.881	344.7 ± 71.3	0.4776	315.7 ± 39.7	0.2535 ± 0.0867	151.9 ± 18.6	0.9697	277.8 ± 197.2	− 9930 ± 9660	257.0 ± 71.0	0.5431
	100	− 2.840 ± 2.935	380.2 ± 72.6	0.3172	309.7 ± 25.6	0.2657 ± 0.1037	245.9 ± 9.5	0.9866	283.3 ± 139.6	− 9876 ± 3835	309.7 ± 50.0	0.7110
	25	− 6.506 ± 4.194	581.0 ± 103.7	0.5445	464.7 ± 42.4	0.4183 ± 0.0368	305.7 ± 27.0	0.9827	290.9 ± 393.9	− 9902 ± 6270	449.4 ± 141.2	0.2450
50	50	− 4.900 ± 4.415	416.5 ± 109.2	0.3795	471.7 ± 72.5	0.2566 ± 0.1624	183.6 ± 28.5	0.9553	438.0 ± 231.1	− 9798 ± 7553	299.2 ± 83.4	0.6827
	100	− 4.262 ± 4.436	372.8 ± 109.7	0.3142	467.4 ± 64.2	0.2658 ± 0.1875	161.7 ± 23.5	0.9635	442.9 ± 190.0	− 9773 ± 8472	265.6 ± 67.2	0.7116

### Determination of the optimal ultrasound time for samples with different volume and preparation amplitudes

**Figure 2:**
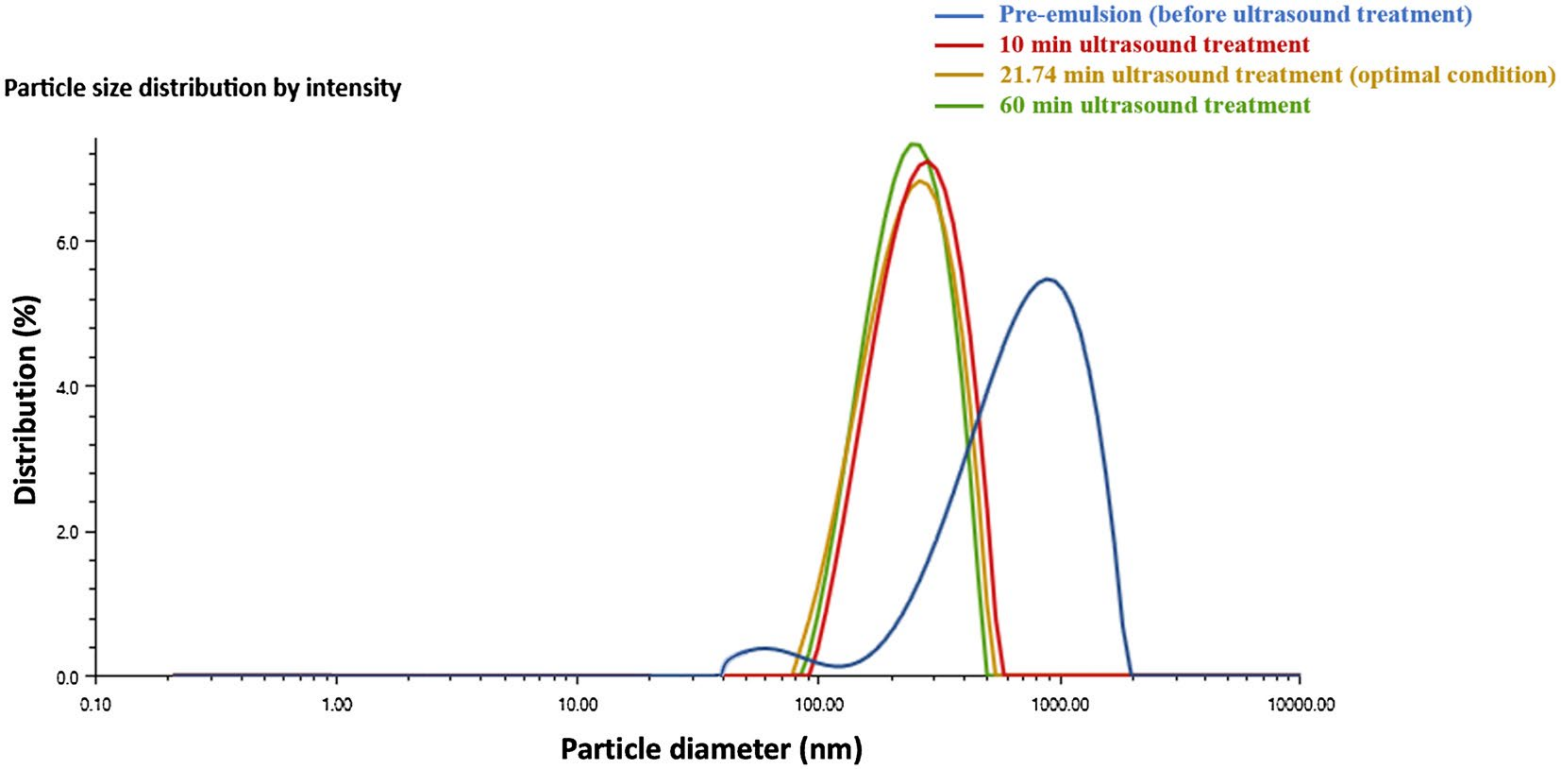
(**a**) Correlation curves between processing time and particle size of samples with different volumes under various ultrasonic amplitudes. (**b**) Correlation curves between T_opt_ and τ % (red shows samples processed at 25% amplitude; green shows samples processed at 50% amplitude; and blue shows samples processed at 100% amplitude).

After confirming that the relationship between the process time and the particle size followed first order, the optimal ultrasound time (T_opt_) was calculated by substituting the optimal particle sizes (PS_opt_) into their regression equations. All samples were regressed according to the first-order model and R^2^ were higher than 0.9 (Fig. 2a). After substituting PS_opt_ into these regression equations, the relationship between the T_opt_ and τ % of all samples were investigated (Fig. 2b). From these curves and Eq. (4), it can be seen that the T_opt_ tended to be the same once the τ % was close to 100%. Thus, in this study, τ % was defined as 99% and used to calculate the optimal ultrasonic preparation times (T_opt_) of each sample based on the first-model. The results are shown in Table 3.

**Table 3:** T_opt_ of samples with different volumes under various amplitudes.

Sample volume	T_opt_25%_ (min)	T_opt_50%_ (min)	T_opt_100%_ (min)	T_opt_average_ (min)
5 ml	10.6 ± 0.8	9.7 ± 0.6	10.6 ± 0.9	10.3 ± 0.5
10 ml	9.5 ± 0.7	10.4 ± 0.5	10.2 ± 0.3	10.0 ± 0.5
20 ml	10.6 ± 0.4	9.3 ± 0.2	9.7 ± 0.8	9.9 ± 0.7
30 ml	10.1 ± 0.1	9.7 ± 0.7	10.4 ± 0.2	10.1 ± 0.4
50 ml	9.9 ± 0.1	10.1 ± 0.6	9.6 ± 0.2	9.9 ± 0.3
500 ml	10.4 ± 0.4	10.1 ± 0.6	10.3 ± 0.5	10.3 ± 0.1

**Figure 3:**
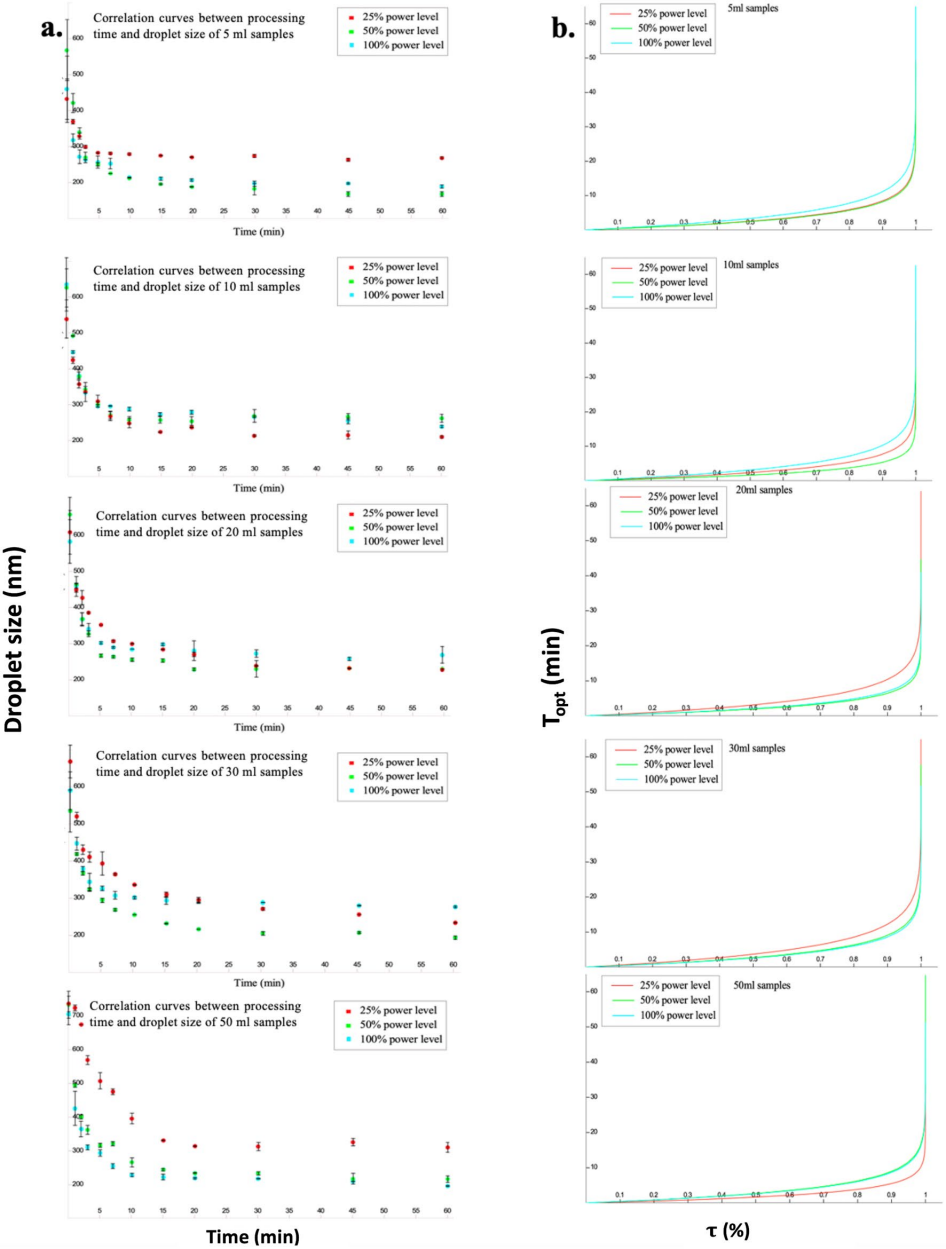
Particle size distribution of emulsion samples with different processing times. (blue shows pre-emulsion before ultrasound processing; red shows emulsion processed for 10 min; yellow shows optimized emulsion processed for 21.74 min; and green shows emulsion processed for 60 min).

Interestingly, it can be shown that the T_opt_ (99%) of all samples in different volumes treated by various amplitudes were all around 10 min. To verify the accuracy of T_opt_ (99%), a sample under the same optimal conditions given by response surface methodology but with 10 min ultrasound treatment was prepared. It showed similar particle size reduction effects as the sample with 21.74 min ultrasound treatment (RSM optimal result) and the sample with 60 min ultrasound treatment (Fig. 3).

In order to prove that T_opt_ can be applied to samples with larger volumes as well, the T_opt_ of 500 ml samples was measured as 10.28 min by the same method (Supplementary Fig. S2; Table 3). Further, in order to verify if T_opt_ can be applied to samples with different oil phase and surfactants, the T_opt_ of another nanoemulsion with olive oil and tween 80 was also tested as 9.93 min by the same method (Supplementary Fig. S3; Table 4). Thus, when probe-type ultrasound processors are used to prepare nanoemulsions, 10 min is enough for reducing the particle size for samples of different volume under various ultrasonic amplitudes.

**Table 4:** First-order regression parameters of the olive oil-tween 80 nanoemulsions with different volumes under various ultrasonic amplitudes, along with their T_opt_.

Model		First-order model					
Volume / mL	Amplitude / %	a	b		c		R^2^
5	25	1200.0 ± 54.1	0.459 ± 54.1		217.0 ± 4.2		0.9486
	50	1241.0 ± 37.1	0.406 ± 0.0945		209.1 ± 15.1		0.9114
	100	1306.0 ± 57.2	0.399 ± 0.1315		242.6 ± 18.8		0.9340
50	25	1348.0 ± 61.0	0.314 ± 0.1159		237.3 ± 29.7		0.966
	50	1104.0 ± 29.4	0.508 ± 0.0811		217.5 ± 11.1		0.9789
	100	1264.0 ± 27.6	0.411 ± 0.0995		187.4 ± 10.5		0.9457
500	25	544.1 ± 42.4	0.720 ± 0.0368		693.5 ± 27.0		0.9794
	50	759.0 ± 72.5	0.664 ± 0.1624		402.5 ± 28.5		0.9331
	100	1005.0 ± 64.2	0.457 ± 0.1875		300.3 ± 23.5		0.9844

T_opt_ for various olive oil-tween 80 nanoemulsions							
Sample volume	T_opt_25%_ (min)	T_opt_50%_ (min)	T_opt_100%_ (min)		T_opt_average_ (min)		
5 ml	9.6 ± 0.4	9.8 ± 0.6	10.4 ± 0.4		9.9 ± 0.5		
50 ml	10.2 ± 0.2	9.5 ± 0.6	10.4 ± 0.8		10.0 ± 0.3		
500 ml	10.9 ± 0.4	9.1 ± 0.7	9.7 ± 0.7		9.9 ± 0.9		

## Discussion

According to the results of the RSM, the hemp seed oil content (%) and lecithin content (%) had a significant effect on particle size (*p* < 0.05), whereas the poloxamer content (%) and the process time (min) did not show significant effect (*p* > 0.05). Based on the sum of squares (Table 1), the relative importance of the respective variables can be ranked as: Lecithin content > Hemp seed oil content > (process time and poloxamer content). The importance of hemp seed oil content is obvious as it was the essential oil phase that can directly impact the particles in the emulsion system, as a larger hemp seed oil content requires a higher amount of surfactant. However, the lecithin concentration more significantly affected the final particle size than the hemp seed oil content due to the highest sum of squares and lowest p-value for the goodness-of-fit test, which underlines the importance of surfactant to obtain smaller particle size, as shown in previous research^1,19^.

Generally, the surfactants with HLB values of 8–16 are considered suitable for O/W emulsion, whereas those with 3–6 HLB are preferable for W/O emulsion^1,19^. Although lecithin shows the HLB value of 4, in a relatively small content in combination with poloxamer, it had a positive impact on particle size reduction in our prepared emulsion systems. However, once its content exceeded a particular content, it started showing a negative impact on particle size reduction (Supplementary Fig. S1a). Whereas, the HLB value for poloxamer is too high (29), and as a result, its individual concentration had no significant effect (*p* < 0.05) on the particle size reduction. It seems that its coupled effect with the lecithin content was the one that established the suitable HLB value (or the surfactant activity). However, it must be noted that at high hemp seed oil content, a higher poloxamer content (%) resulted in increased particle sizes (nm), which implied that the interaction effect of the two variables had a negative impact on the particle size reduction. These observations led to the conclusion that the content of the hemp seed oil and the surfactant should be confirmed before studying the relationship between the particle size reduction and the ultrasonication process time.

Both individual and mutual interactions of process time had no significant effect (*p* < 0.05) on the particle size, indicating that the final particle size (nm) was only dependent on the system (i.e. the concentrations of the oil and aqueous phases and the surfactants). This suggests the possibility of determination of an optimal process time which yields almost the same particle size reduction as a process continuing for an infinite time. Thereby, the particle reducing behavior was characterized by fitting it to well-known mathematical models to confirm whether it was necessary to keep increasing process time to get the smallest particle size.

**Figure 4:**
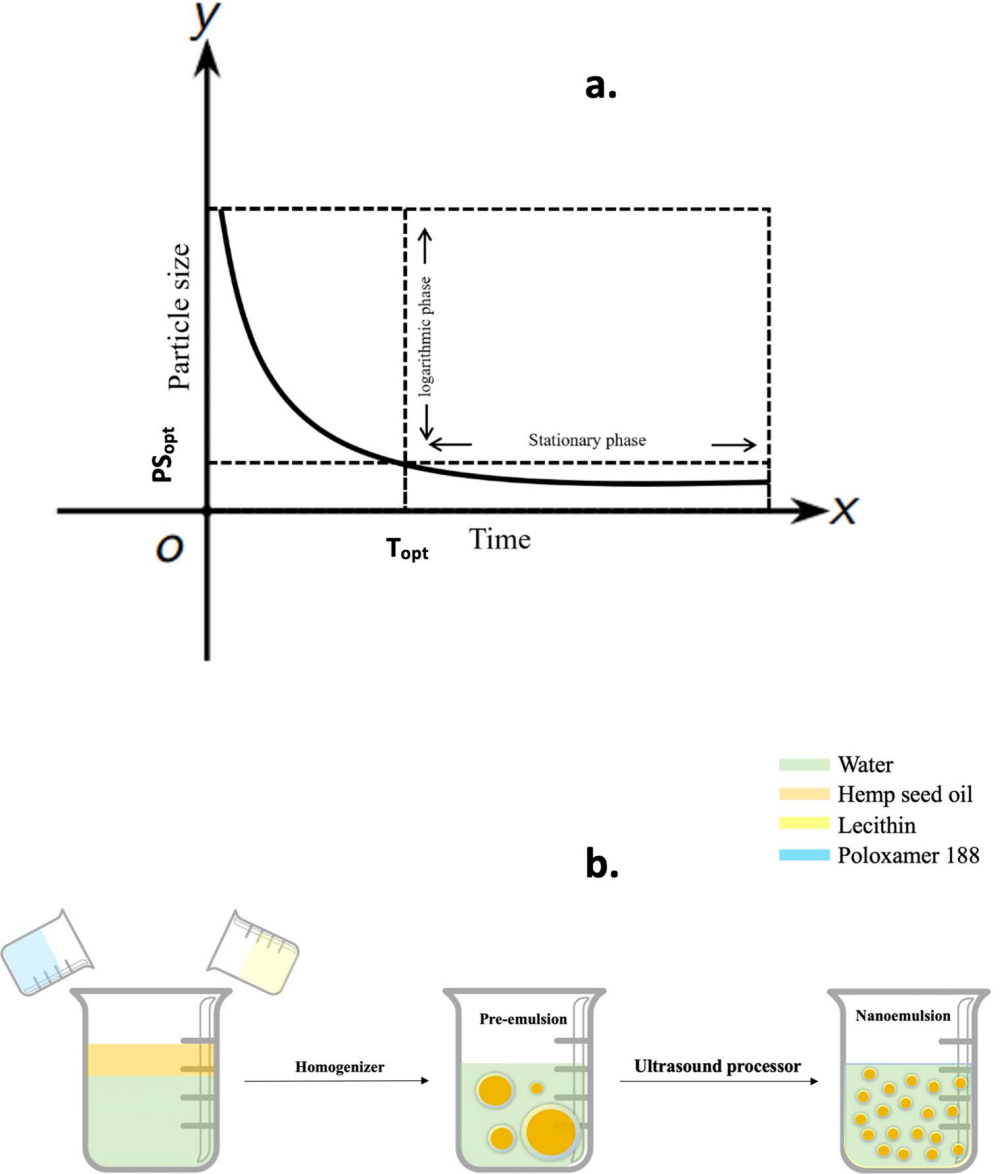
(**a**) Optimal nanoemulsion characterization, (**b**) Processing steps of the hemp seed oil nanoemulsion**.**

After they were fitted into zero-order, first-order and second-order mathematical models, the first-order model was found to be best to modulate the relationship between processing time and particle size reduction. Generally, the first-order model consists of two parts, the logarithmic phase and stationary phase (Fig. 4a). The horizontal ordinate to the turning point of these two phases can be considered the optimal ultrasonic preparation times (T_opt_) because increasing the ultrasonic time beyond this time will not have much effect on the particle size reduction. This implies that an optimal ultrasonic preparation time can be suggested for the nanoemulsion system. In this study, T_opt_ was defined as the optimal process time which will give τ % of the particle size reduction as that of an infinitely long process (Fig. 4a). After substituting PS_opt_ into these regression equations, the relationship between the T_opt_ and τ % of all these samples were also established (Fig. 2b). The relationship between the T_opt_ and τ % was given as Eq. (2).

2 $$Topt = \frac{-2.303}{b} \times log\left(\frac{\left(1-\tau \%\right) PSbefore + \tau \% PSafter -c}{a}\right)$$

As the the relationship between the processing time and the particle size follows the first order:

3 $$Ps\left(t\right)=a{e}^{-b*t}+c$$

when t → ∞, $Ps\left(t\right)\to c$. Thus, *c* means the final particle size after ultrasonic processing after very long time of processing (1 h in our case). When t = 0, $Ps\left(t\right)=\mathrm{a}+c$. Therefore, *a* is the maximum particle size reduction possible by the ultrasonic processing. Accordingly, the PS_before_ and the PS_after_ can be written as $\mathrm{a}+c$ and $c$, respectively. After $\mathrm{a}+c$ and $c$ were put into Eq. (2), the relationship between the T_opt_ and τ % became Eq. (4)

4 $$Topt = \frac{-2.303}{b} \times log\left(1-\tau \%\right).$$

From Eq. (4) and its regression curves (Fig. 2a), it can be seen that the T_opt_ tended to be the roughly same, once the τ % was close to 1. Thus, τ % was defined as 99% in this study and was used to calculate the optimal ultrasonic preparation times (T_opt_) of each sample based on the first-model. The T_opt_ were all around 10 min amongst different volume samples treated with various amplitudes. In addition, since in our study, the relationship between process time and particle size reduction was only fitted into zero-order, first-order and second-order models, the nth-order model should also be discussed. The nth-order model and its integrated form can be given as Eq. (5) and Eq. (6) respectively.

5 $$-\frac{d[A]}{dt} = k{[A]}^{n}$$

6 $$\frac{1}{{[A]}^{n-1}} = \frac{1}{{{\left[A\right]}_{0}}^{n-1}} + (n-1)kt$$

In our case, the relationship between process time and particle size (Ps (t)) reduction can be written as Eq. (7).

7 $$Ps\left( t \right) = \left( {\frac{1}{{\frac{1}{a} + \left( {n - 1} \right) bt}}} \right)^{{\frac{1}{n - 1}}} + c$$

In this equation, when t → ∞, $Ps\left(t\right)\to c$. Thus, *c* means the final particle size after ultrasonic processing. When t = 0, $Ps\left(t\right)={a}^{\frac{1}{n-1}}+c$. Therefore, ${a}^{\frac{1}{n-1}}+c$ is the maximum particle size reduction possible by the ultrasonic processing and the PS_opt_ can be written as PS_opt_ = ${a}^{\frac{1}{n-1}}+c-$ $\tau \%\left( {a}^{\frac{1}{n-1}}\right)$. After PS_opt_ was substituted into Eq. (7), the relationship between the T_opt_ and τ % can be given as Eq. (8)

8 $$Topt = \frac{1-{(1-\tau \%)}^{n-1}}{ab(n-1){(1-\tau \%)}^{n-1}}$$

Based on previous study^10^ and our case (Fig. 2), the total particle size reduction was insensitive to ultrasonication amplitude and volume. The time scale (b), which was the slope of each curve, also tended to be similar (Table 2). It is also highly related to Weber number, which indicates the ratio of inertial to surface tension force. In our case, as the ratio of the oil and water phases were similar, so the the value of b was uniquely related to the Weber number. Accordingly, T_opt_ was found to be similar among different samples regardless of the emulsion volume and ultrasonic amplitude.

What’ s more, it can be seen from the results that higher ultrasonic amplitude could not always have better effects to reduce the particle sizes of samples with various volumes, which was consistent with the previous research, which was consistent with the previous research^10^ (Fig. 2). For samples of 5 ml, 20 ml and 30 ml, 50% of full power reduced the particle size the most in one-hour testing time. In contrast, 25% of full power was more suitable for 10 ml sample and 100% of full power was the best for 50 ml and 500 ml samples (Fig. 2). Based on previous study^25^, the bubbles in the region of average cavitation intensity created by ultrasound underwent a relatively less violent collapse than the bubbles in the region of the highest cavitation intensity because of their lower expansion. Accordingly, when the amplitude was too high, the droplets tended to collide and then combined to form larger droplets. Therefore, for minimizing particle size, it is better to select the ultrasonic amplitude as one factor to optimize the preparation of the nanoemulsions instead of processing time.

## Conclusion

The significance of this study is to present the existence of a constant optimal ultrasonic preparation time for nanoemulsions preparation. Ultrasound-based nanoemulsion was attempted using two surfactants to encapsulate hemp seed oil and their optimal contents were obtained by response surface methodology to produce the smallest particle size. The results indicated that the combination of 12.5% (v/v) hemp seed oil, 2.6% (v/v) poloxamer 188 and 5.9% (w/v) lecithin was most suitable for this nanoemulsion system, resulted in 176.4 nm of particle size, 0.239 of PDI, 31.4 mv of zeta potential and 96.08% of entrapment efficiency. It was also showed that the particle size reduction was not significantly impacted (*p* > 0.05) by the processing time. Besides, in this study, the first-order model was found the best to modulate the relationship between the processing time and the particle size reduction. The applicability of the first-order model suggests it is not necessary to keep increasing processing time to get the smallest particle size because increasing the ultrasonic time does not have much effect on the particle size reduction after specific time (T_opt_). The T_opt_ was calculated by substituting the optimal particle sizes (PS_opt_) into the regression equations of samples with various volumes treated with different ultrasonic amplitudes within one hour. Interestingly, the T_opt_ of all the samples was around 10 min, no matter their volumes (mL) and the amplitudes (%) used for processing. To prove the validity of the T_opt_, a sample was prepared under the same optimal conditions given by the RSM but with 10 min ultrasound treatment. The results showed no significant difference (*p* > 0.05) between the particle size of the prepared sample and that of the optimal sample. To expand the scope of application, samples with 500 mL volumes and different oil phases and surfactants were also tested for their T_opt_ and they all showed similar results. Thus, using an ultrasound probe-type processor for 10 min was enough for reducing the particle size of samples in different volumes under various ultrasonic amplitudes. To apply these results to the n^th^-order model, the size difference between the pre-emulsions and the final nanoemulsions was found to be one crucial factor for nanoemulsion size reduction. It is noteworthy that in this study, the particle sizes of all the pre-emulsions before ultrasound treatment were around 1000 nm. Since probe-type ultrasound processors might not be capable of generating sufficient intense disruptive forces in a very unhomogenized medium^26^, it is better to reduce the particle size of pre-emulsion to 1000 nm before the 10-min ultrasound treatment. What’s more, it can be seen from the results that higher ultrasonic amplitude could not always have better effects to reduce the particle size so that it is better to select the ultrasonic amplitude as one factor to optimize the preparation of the nanoemulsion instead of the preparation time.

## Materials and methods

### Materials

Unrefined cold-pressed hemp seed oil (HSO) was purchased from Manitoba Harvest Hemp Foods (Winnipeg, Manitoba, Canada). Olive oil was purchased from Compliments (Mississauga, Ontario, Canada). Refined lecithin was purchased from Alfa Aesar Co. Inc (Mississauga, Ontario, Canada). Poloxamer 188 was obtained from Corning Co. Inc (Manassas, Virginia, USA). Tween 80 was from Fisher Scientific (Waltham, Massachusetts, USA). Methanolic potassium hydroxide solution (2 M), HCL (2 M) and heptane were purchased from Sigma-Aldrich Canada Corp. (Oakville, Ontario, Canada) and were of analytical or chromatography grade.

### Optimization of the preparation condition of the nanoemulsions

#### Nanoemulsion preparation

A two-step process was employed to prepare the O/W nanoemulsions (Fig. 4b). All pre-emulsions were prepared by a Polytron PCU-2-110 homogenizer (Brinkmann Ind. Westbury, NY, USA). For the CCRD design, the preparation process is described as follows: HSO was mixed with distilled water in desired concentrations as shown in Table 1 to obtain a 50 ml solution. Then the different concentration of lecithin and poloxamer 188 (shown in Table 1) were added into this solution and homogenized for 10 min at a speed setting of half-maximum (= 5). Then the O/W nanoemulsions were formed by ultrasonicating (UP 200ST, Hielscher Ultrasonics, Teltow, Germany) the pre-emulsions for different time using 50% of full power. For the kinetic analysis employed, 5, 10, 20, 30 and 50 ml pre-emulsions were prepared by using HSO, lecithin and polaxamer 88 in the optimal ratio calculated from the CCRD design, using Polytron homogenizer, as described above. Samples were then ultrasonicated at 25, 50 and 100% power levels for upto 1 h. For verification with olive-oil samples, one other nanoemulsion was prepared using olive oil and tween 80 with the same method. To control the temperature (and thus isolate its’ effects) during the ultrasonication process, all samples were kept in ice-bath. All the nanoemulsions were stored at 4 °C for further tests.

#### Response surface optimization

A central composite rotatable design (CCRD) was selected for process optimization using Minitab software version 18.0 (Minitab LLC, State College, PA, USA) and Design Expert version 11 (Stat-Ease Inc., Minneapolis, MN, USA). The variables were HSO content (% v/v), poloxamer content (% v/v), lecithin content (% w/v) and ultrasound processing time (min) at five coded levels (− *α*, − 1, 0, + 1, + *α*). They were generated by the software, as shown in Table 1. The central composite design had 30 experimental trials which included 16 trials for the factorial design, 8 trials for axial points (2 for each variable) and 6 trials for replications of the central points. The responses were fitted to a non-linear model, as shown in Eq. (9).

9 $$\begin{aligned} {\text{Y}} & = \beta_{0} + \beta_{1} {\text{A}} + \beta_{2} {\text{B }} + \beta_{3} {\text{C}} + \beta_{4} {\text{D }} + \beta_{1}^{2} {\text{A}}^{{2}} + \beta_{2}^{2} {\text{B}}^{{2}} + \beta_{3}^{2} {\text{C}}^{{2}} + \beta_{{4}}^{{2}} {\text{D}}^{{2}} + \beta_{{1}} \beta_{{2}} {\text{AB}} \\ & \quad + \beta_{1} \beta_{3} {\text{AC}} + \beta_{1} \beta_{4} {\text{AD }} + \beta_{2} \beta_{3} {\text{BC}} + \beta_{2} \beta_{4} {\text{BD }} + \beta_{3} \beta_{4} {\text{CD}} \\ \end{aligned}$$

where Y was the response variable, *β*_0_ was the intercept term, *β*_1_–*β*_4_ were linear coefficients, *β*_1_* β*_2_–*β*_3_* β*_4_ were the interactive coefficients, *β*_1_^2^ – *β*_4_^2^ were the quadratic coefficients and A, B, C, D represent the independent variables. Low and high factor settings were coded as − 1 and + 1. The central point was coded as 0. All samples prepared were 50 ml. The fitted Eq. (9) was optimized for minimizing process time.

### Emulsion characterization

The hydrodynamic diameters of the emulsion droplets were obtained with a dynamic light scattering (DLS) using Litesizer 500 (Anton Paar, Graz, Austria). The measurements were performed using automatic settings adjusted for the latex spheres at 25 °C. Zeta potential was determined using Omega cuvette Z (Anton Paar, Graz, Austria) with Litesizer 500 (Anton Paar, Graz, Austria) at 25 °C. The morphology of the nanoemulsions was observed by FEI Tecnai G2 Twin Transmission electron microscopy (TEM) (FEI, Hillsboro, United States) using cryomode. The images were later analyzed using FEI Xpress 3D software (FEI, Hillsboro, United States).

### Entrapment efficiency

Entrapment efficiency of the nanoemulsions was determined according to previous research^18^. It was found that linoleic acid (LA) is the crucial fatty acid in hemp seed oil^18^ so that it can be used as a marker for analyzing the entrapment efficiency. For this purpose, the prepared samples were centrifuged at 12,500 rpm at 20 °C for 15 min to precipitate the particles in the emulsion system. After the centrifugation, the untrapped hemp seed oil was collected from the supernatant. The fatty acid percentages including LA in the supernatant and in the original hemp seed oil were determined by a Shimadzu GC-17A Gas Chromatograph system (Shimadzu, Scientific Instruments, Inc., Columbia MD) equipped with a flame ionization detector using a fused silica capillary column (Omegawax 320, 30 m × 0.32 mm ID × 0.25 µm film thickness). The initial temperature of the column was set at 165 °C for 10 min followed by increasing to 200 °C with a rate of 1.5 °C/min. The entrapment efficiency (EE) of linoleic acid (LA) concentration were determined by comparing the amount of unencapsulated LA (i.e. in the supernatant) with the initial amounts of LA in hemp seed oil as shown in Eq. (10), wherein the amounts of unencapsulated LA and total LA were calculated based on the chromatogram peak area.

10 $$Entrapment\;efficiency~\left( \% \right) = \left({{1 - Unencapsulated\;LA}\mathord{\left/ {\vphantom {{1 -Unencapsulated\;LA}{Total\;LA}}}\right. \kern-\nulldelimiterspace}{Total\;LA}}\right) \times 100\%$$

### Models for the relationship between particle size and ultrasound time

In order to determine the mathematical models followed by the relationship between particle size and ultrasound time, samples with different volumes were prepared under different energy levels for various durations. The contents of hemp seed oil, poloxamer 188 and lecithin were given by RSM results (Figure S1). Pre-emulsions were prepared using the method described in the section of Nanoemulsion preparation. Then, 5, 10, 20, 30 and 50 ml pre-emulsions were subjected to ultrasound for 1, 2, 3, 5, 7, 10, 15, 30, 45 and 60 min under 25%, 50% and 100% of full power, respectively. After preparations, all these emulsions were tested for their particle sizes. The correlation curves between processing times and particle sizes were fitted to zero-order, first-order, and second-order models by plotting the particle size with time using the Matlab Curve fitting tool (R2015a, Mathworks Inc., Natick, MA, USA).

A zero-order linear model expressed by Eq. (11):

11 $$Ps\left(t\right)=at+b$$

A first-order non-linear model expressed by Eq. (4):

12 $$Ps\left(t\right)=a{e}^{-b*t}+c$$

A second-order model adjusted to the following non-linear Eq. (13):

13 $$Ps\left(t\right)=\frac{1}{\frac{1}{a}+(b*t)} +c$$

where *Ps* corresponds to the particle size at any specific time *t*. All the tests were done in triplicate.

### Determination of the optimal ultrasound time for samples with different volume and ultrasonic amplitudes

In order to determine the suggested optimal ultrasonic preparation time (T_opt_), the relationship curves made in previous section were constructed by Microsoft Excel (Ver. 16.16.11, Microsoft Excel for Mac, Microsoft) and the R^2^ of the various fitting equations were used to assess the significance of fit. The most appropriate model (later found out to be the first order model shown in Table 2) was used for calculating T_opt_, by substituting the optimal particle size (PS_opt_) into the appropriate regression equation. PS_opt_ and T_opt_ were defined as the particle size and the corresponding time respectively that result in τ % of the maximum particle size reduction possible after a very long treatment (Eq. 14).

14 $${\text{PS}}_{{{\text{opt}}}} = {\text{ PS}}_{{{\text{before}}}} - \tau \% ({\text{PS}}_{{{\text{before}}}} - {\text{PS}}_{{{\text{after}}}} )$$

where PS_before_ is the particle size of the pre-emulsion before ultrasound treatment and PS_after_ was the particle size after a very long ultrasonic treatment of one hour.

To verify that the T_opt_ was independent of the sample volume, oil phase and surfactant, a larger sample volume of 500 ml and one other nanoemulsion system with different oil phase and surfactant (olive oil and tween 80) were also prepared using the same method and tested for their T_opt_.

### Statistical analysis

All experiments were performed in triplicates and the mean values of particle size distribution and zeta potential were calculated. Regression analysis and analysis of variance (ANOVA) were carried out using Design Expert (Stat-Ease Inc., Minneapolis, Minnesota, USA) and Minitab software version 18.0 (Minitab LLC, State College, PA, USA) to evaluate the level of significance of all the variables and their interactions. R^2^ (coefficient of determination) was used to determine the fitness of different mathematical models using Matlab Curve Fitting Tool (R2015a, Mathworks Inc., Natick, MA, USA).

## Supplementary Information

Supplementary Information

## Data Availability

All data generated or analysed during this study are included in this published article (and its Supplementary Information files).
