# Supplementary material for: Optimal ultrasonication process time remains constant for a specific nanoemulsion size reduction system
Source: Sci Rep. 2021 Apr 29;11:9241. doi: 10.1038/s41598-021-87642-9 (PMC8085214; doi:10.1038/s41598-021-87642-9)
Supplement: Supplementary file 1 — Supplementary Information [file 41598_2021_87642_MOESM1_ESM.docx]

**Optimal Ultrasonication Process Time remains constant for a specific nanoemulsion size reduction system**

Anubhav Pratap-Singh^1,+,*^, Yigong Guo^1,+^, Sofia Lara Ochoa^2^, Farahnaz Fathordoobady^1^, Anika Singh^1^

^1^Faculty of Land and Food Systems (LFS), The University of British Columbia, Vancouver Campus 213-2205 East Mall, Vancouver, BC Canada V6T 1Z4

^2^Department of Chemistry and Nanotechnology, Monterrey Institute of Technology and Higher Education (ITESM), Monterrey Campus, Av. Eugenio Garza Sada 2501 Sur, Tecnologico, 64849 Monterrey, N.L., México

^+^ Authors contributed equally and are listed alphabetically, and are co-first authors

* Corresponding Author: Anubhav Pratap-Singh
Affiliation: Faculty of Land and Food Systems (LFS), University of British Columbia, Vancouver Campus 213-2205 East Mall, Vancouver, BC Canada V6T 1Z4
E-mail Address: [anubhav.singh@ubc.ca](mailto:anubhav.singh@ubc.ca)

**
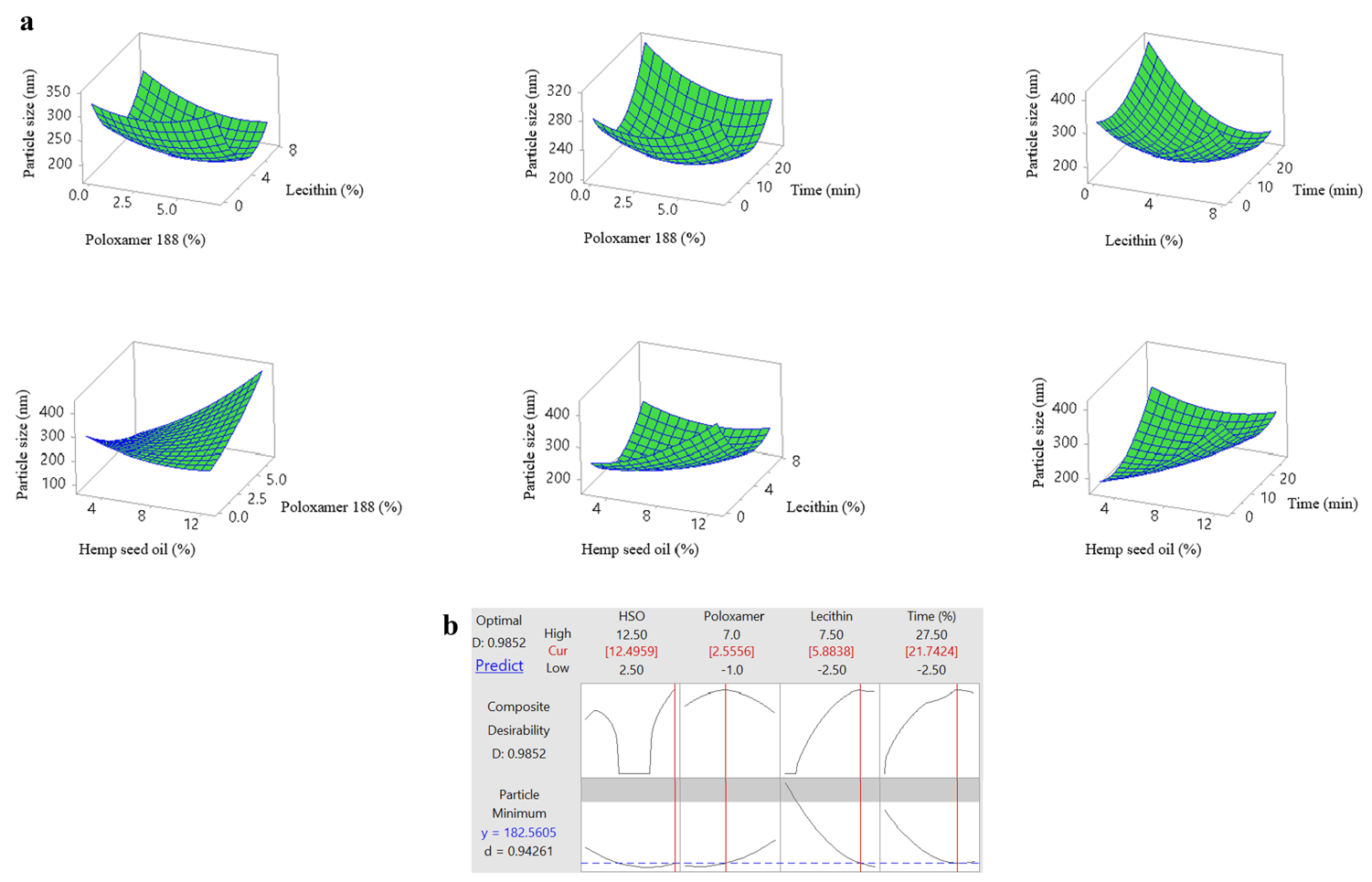
**

**Supplementary Figure S1.** Response surface optimization. (a) 3-D response surface plots showing various factors on particle size. (b) Predicted values of the optimal plots

**
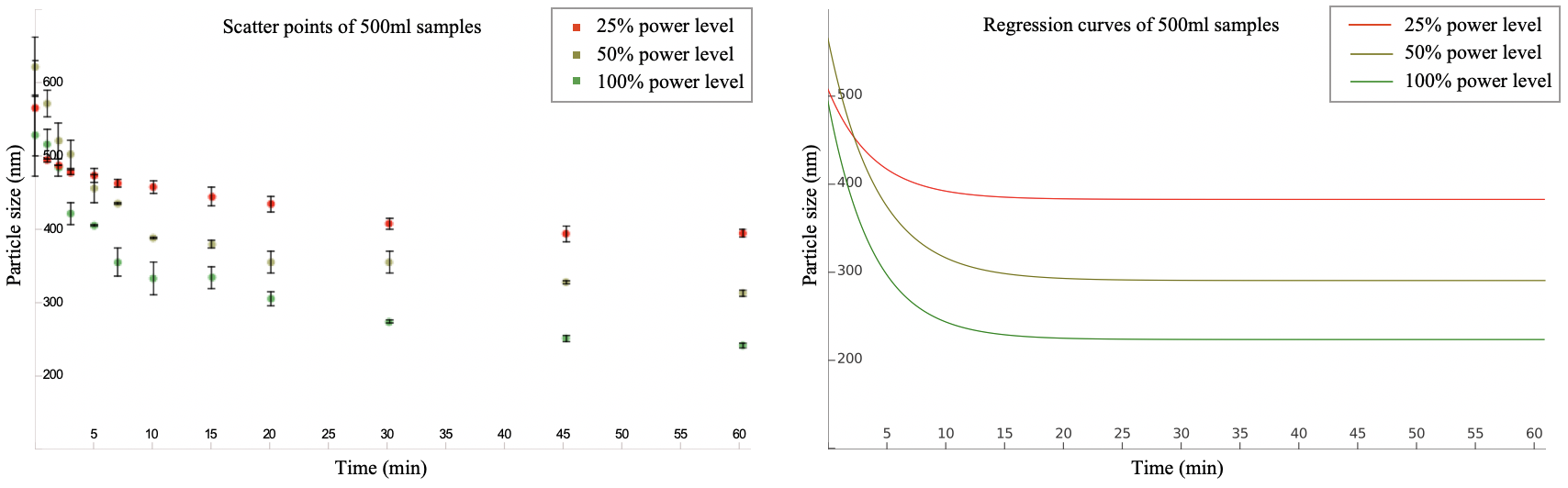
**

**Supplementary Figure S2.** Correlation curves between processing time and particle size of 500 ml samples under various ultrasonic amplitudes (red shows samples processed at 25% amplitude; green shows samples processed at 50% amplitude; and blue shows samples processed at 100% amplitude).


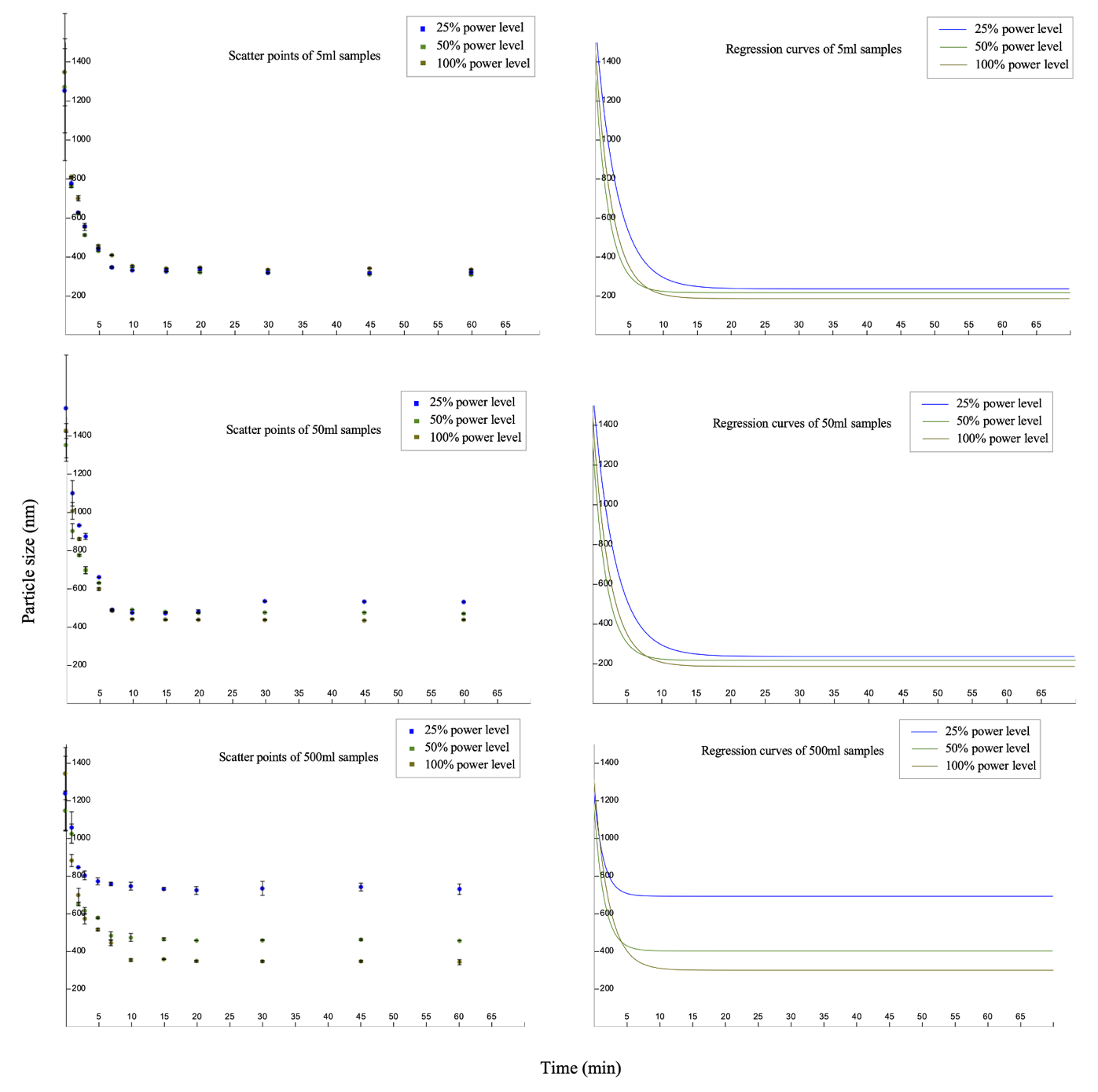


**Supplementary Figure S3.** The correlation curves between processing time and particle size of olive oil-tween 80 nanoemulsions with different volumes under various ultrasonic amplitudes

**Supplementary Table S1.** The raw data of the 5 ml sample treated with different processing time and power levels (all tests were done in triplicate)

| Processing time (min) | Particle Size (nm)  25% power level | | | Particle Size (nm)  50% power level | | | Particle Size (nm)  100% power level | | |
| --- | --- | --- | --- | --- | --- | --- | --- | --- | --- |
| **0** | 505.4 | 433.1 | 391.0 | 668.6 | 573.6 | 496.2 | 457.1 | 385.1 | 570.8 |
| **1** | 372.0 | 386.2 | 380.1 | 423.6 | 462.2 | 409.9 | 304.6 | 338 | 340 |
| **2** | 350.2 | 335.2 | 331.2 | 348.8 | 363.5 | 337.3 | 304.3 | 266.8 | 272.1 |
| **3** | 302.6 | 309.0 | 313.3 | 293.6 | 283.6 | 261.3 | 272.2 | 272.6 | 273.3 |
| **5** | 289.6 | 291.0 | 296.5 | 254.7 | 265.4 | 258.9 | 258.3 | 252.4 | 286.9 |
| **7** | 289.2 | 295.4 | 286.5 | 235.5 | 234.3 | 234.3 | 265 | 276.1 | 244.8 |
| **10** | 283.5 | 292.8 | 289.3 | 219.7 | 221.0 | 221.6 | 223.4 | 222.4 | 224.1 |
| **15** | 284.7 | 284.7 | 283.7 | 207.6 | 203.4 | 202.6 | 214.6 | 220.7 | 224.7 |
| **20** | 277.8 | 279.9 | 281.6 | 194 | 198 | 198.8 | 216.3 | 219.7 | 211.2 |
| **30** | 284.7 | 289.1 | 276.9 | 203.8 | 198 | 172 | 200.4 | 215.9 | 202.1 |
| **45** | 276.0 | 275.7 | 266.9 | 172.6 | 173.9 | 185.3 | 203 | 209.8 | 205.9 |
| **60** | 281.9 | 273.0 | 278.0 | 173.2 | 171.8 | 185.5 | 202.7 | 199.8 | 191.6 |

**Supplementary Table S2** The raw data of the 10 ml sample treated with different processing time and power levels (all tests were done in triplicate)

| Processing time (min) | Particle Size (nm)  25% power level | | | Particle Size (nm)  50% power level | | | Particle Size (nm)  100% power level | | |
| --- | --- | --- | --- | --- | --- | --- | --- | --- | --- |
| **0** | 625.3 | 506.2 | 569.7 | 725.3 | 606.2 | 659.7 | 582.4 | 747.3 | 691.8 |
| **1** | 453.6 | 431.5 | 439.1 | 518.7 | 512.8 | 515.5 | 459.9 | 469.1 | 467.2 |
| **2** | 380.0 | 353.6 | 368.4 | 370.9 | 383.2 | 418.9 | 402.7 | 396.5 | 381.4 |
| **3** | 368.7 | 310.4 | 353.4 | 355.9 | 338.6 | 358 | 342.1 | 341.4 | 340 |
| **5** | 330.2 | 320.2 | 293.1 | 311.8 | 310.2 | 294.5 | 304 | 301.2 | 294.6 |
| **7** | 253.1 | 276.5 | 276.7 | 265.5 | 263.1 | 288.2 | 302.8 | 298.8 | 297.6 |
| **10** | 248.4 | 231.1 | 262.7 | 246.3 | 254.8 | 270.1 | 292 | 296.8 | 282.2 |
| **15** | 220.5 | 221.0 | 220.3 | 261.9 | 265.6 | 244.7 | 268.9 | 273.1 | 282.5 |
| **20** | 232.3 | 236 | 235.2 | 252 | 269.1 | 239.3 | 273.8 | 285.1 | 285.5 |
| **30** | 208.7 | 205.7 | 212.0 | 260.4 | 293.3 | 255.4 | 268.3 | 263.2 | 269.3 |
| **45** | 212.3 | 222.9 | 196.8 | 263.7 | 280.3 | 259.3 | 260.2 | 259.9 | 243.6 |
| **60** | 207.5 | 209.1 | 199.5 | 267.5 | 246.6 | 273.2 | 235.3 | 242.4 | 233.8 |

**Supplementary Table S3** The raw data of the 20 ml sample treated with different processing time and power levels (all tests were done in triplicate)

| Processing time (min) | Particle Size (nm)  25% power level | | | Particle Size (nm)  50% power level | | | Particle Size (nm)  100% power level | | |
| --- | --- | --- | --- | --- | --- | --- | --- | --- | --- |
| **0** | 640.5 | 551.2 | 681.7 | 627.3 | 672.4 | 731.8 | 522.3 | 649.3 | 619.3 |
| **1** | 473.5 | 432.5 | 457.1 | 463.5 | 450.4 | 497.1 | 457.8 | 471.0 | 469.8 |
| **2** | 429.3 | 453.3 | 409.6 | 389.5 | 360.5 | 353.3 | 390.5 | 362.5 | 348.4 |
| **3** | 382.1 | 390.4 | 387.0 | 333.2 | 323.8 | 315.9 | 355.6 | 338.1 | 320.7 |
| **5** | 352.3 | 349.7 | 350.1 | 254.7 | 265.4 | 258.9 | 302.5 | 292.5 | 295.8 |
| **7** | 306.3 | 295.8 | 305.2 | 257.3 | 251.6 | 260.6 | 280.4 | 284.0 | 286.6 |
| **10** | 294.9 | 296.3 | 291.3 | 248.9 | 251.6 | 241.3 | 278.4 | 279.4 | 276.7 |
| **15** | 278.1 | 280.8 | 275.0 | 250.2 | 239.4 | 245.7 | 289.2 | 298.2 | 290.4 |
| **20** | 272.2 | 259.6 | 257.8 | 224.3 | 217.3 | 214.3 | 284.2 | 298.7 | 239.8 |
| **30** | 228.0 | 231.7 | 228.1 | 206.0 | 249.3 | 205.4 | 255.7 | 262.2 | 279.2 |
| **45** | 219.4 | 223.8 | 223.7 | 225.2 | 221.1 | 220.4 | 250.6 | 255.8 | 244.6 |
| **60** | 221.2 | 217.5 | 214.7 | 221.4 | 220.7 | 218.8 | 242.5 | 290.6 | 251.2 |

**Supplementary Table S4** The raw data of the 30 ml sample treated with different processing time and power levels (all tests were done in triplicate)

| Processing time (min) | Particle Size (nm)  25% power level | | | Particle Size (nm)  50% power level | | | Particle Size (nm)  100% power level | | |
| --- | --- | --- | --- | --- | --- | --- | --- | --- | --- |
| **0** | 628.1 | 724.4 | 672.9 | 473.8 | 534.7 | 596.1 | 647.0 | 594.6 | 537.6 |
| **1** | 522.5 | 530.3 | 505.2 | 407.0 | 414.4 | 415.7 | 449.5 | 458.2 | 421.2 |
| **2** | 437.4 | 427.3 | 409.3 | 352.4 | 358.5 | 364.8 | 366.5 | 381.1 | 363.7 |
| **3** | 401.0 | 389.6 | 422.5 | 309.9 | 319.6 | 308.6 | 363.4 | 321.0 | 316.3 |
| **5** | 365.6 | 365.7 | 425.9 | 287.2 | 273.5 | 285.2 | 321.7 | 315.6 | 306.7 |
| **7** | 357.3 | 351.4 | 356.6 | 259.5 | 250.0 | 253.4 | 294.2 | 282.7 | 307.4 |
| **10** | 322.0 | 327.1 | 327.2 | 241.5 | 240.1 | 238.4 | 286.1 | 286.9 | 294.3 |
| **15** | 305.3 | 293.9 | 294.5 | 216.1 | 216.7 | 213.5 | 286.5 | 287.0 | 267.6 |
| **20** | 285.1 | 287.8 | 271.5 | 197.1 | 200.1 | 200.3 | 284.5 | 279.1 | 270.3 |
| **30** | 253.9 | 255.4 | 261.3 | 181.2 | 190.2 | 192.6 | 275.4 | 272.4 | 276.1 |
| **45** | 241.9 | 241.9 | 239.2 | 192.8 | 190.3 | 184.9 | 268.7 | 266.3 | 264.0 |
| **60** | 219.5 | 216.9 | 215.9 | 168.7 | 179.3 | 178.3 | 260.5 | 260.4 | 266.2 |

**Supplementary Table S5** The raw data of the 50 ml sample treated with different processing time and power levels (all tests were done in triplicate)

| Processing time (min) | Particle Size (nm)  25% power level | | | Particle Size (nm)  50% power level | | | Particle Size (nm)  100% power level | | |
| --- | --- | --- | --- | --- | --- | --- | --- | --- | --- |
| **0** | 717.2 | 738.4 | 765.1 | 778.5 | 738.4 | 694.6 | 713.8 | 738.9 | 672.7 |
| **1** | 718.5 | 725.3 | 738.9 | 488.8 | 504.4 | 493.6 | 485.8 | 406.9 | 384.9 |
| **2** | 676.8 | 678.1 | 678.5 | 402.3 | 408.2 | 392.3 | 341.7 | 389.9 | 360.4 |
| **3** | 557.3 | 572.2 | 584.3 | 355.4 | 352.0 | 377.1 | 302.5 | 319 | 307.5 |
| **5** | 537.1 | 488.6 | 498.5 | 321.5 | 316.1 | 307.6 | 297.4 | 298.3 | 280.2 |
| **7** | 476.0 | 467.2 | 485.8 | 313.5 | 319.1 | 329.1 | 244.7 | 250.9 | 263.1 |
| **10** | 415.1 | 390 | 380.7 | 281.2 | 253.1 | 258.6 | 228.8 | 217.9 | 230.0 |
| **15** | 331.7 | 327.7 | 329.8 | 247.2 | 241.2 | 238.5 | 221.6 | 209.2 | 228.1 |
| **20** | 314.3 | 309.7 | 313.8 | 229.6 | 234.0 | 230.1 | 212.7 | 217.4 | 220.6 |
| **30** | 305.7 | 302.2 | 326.4 | 238 | 226.1 | 228.9 | 212.2 | 215.5 | 217.3 |
| **45** | 337.7 | 312.8 | 322.4 | 203.7 | 234.2 | 204.9 | 206.9 | 205.7 | 208.8 |
| **60** | 309.2 | 324.0 | 293.3 | 201 | 217.3 | 223.4 | 195.6 | 190.9 | 192.2 |

**Supplementary Table S6** The raw data of the 500 ml sample treated with different processing time and power levels (all tests were done in triplicate)

| Processing time (min) | Particle Size (nm)  25% power level | | | Particle Size (nm)  50% power level | | | Particle Size (nm)  100% power level | | |
| --- | --- | --- | --- | --- | --- | --- | --- | --- | --- |
| **0** | 635.6 | 558.6 | 505.0 | 596.4 | 670.4 | 600.7 | 474.6 | 588.0 | 525.1 |
| **1** | 495.1 | 498.5 | 492.3 | 566.1 | 556.8 | 594.1 | 515.9 | 495.6 | 538.8 |
| **2** | 486.8 | 488.1 | 488.2 | 508.4 | 505.5 | 550.7 | 498.1 | 481.2 | 475.0 |
| **3** | 481.0 | 480.1 | 473.5 | 519.3 | 509.3 | 481.1 | 427.6 | 405.0 | 433.8 |
| **5** | 473.5 | 485.5 | 463.3 | 438.3 | 452.9 | 478.6 | 407.3 | 404.0 | 405.6 |
| **7** | 456.9 | 466.0 | 467.3 | 436.7 | 432.5 | 437.9 | 378.3 | 345.8 | 341.8 |
| **10** | 451.3 | 468.2 | 455.8 | 386.9 | 387.4 | 391.9 | 310.7 | 331.9 | 357.4 |
| **15** | 433.6 | 440.2 | 461.0 | 386.6 | 375.5 | 377.9 | 338.8 | 348.0 | 317.7 |
| **20** | 447.0 | 434.6 | 424.3 | 370.7 | 339.9 | 355.9 | 308.3 | 314.7 | 294.0 |
| **30** | 398.5 | 414.5 | 411.7 | 355.9 | 339.9 | 370.7 | 276.7 | 274.2 | 270.5 |
| **45** | 387.3 | 408.2 | 388.2 | 325.8 | 330.9 | 328.2 | 254.2 | 245.9 | 254.6 |
| **60** | 392.2 | 391.6 | 401.7 | 307.6 | 313.9 | 318.9 | 246.1 | 238.2 | 241.4 |
